# Atlas of Methods of Clinical Investigation

**Published:** 1904-01

**Authors:** 


### Books and Magazines

ATLAS OF METHODS OF CLINICAL INVESTIGATION, with an Epitome of Clinical Diagnosis and of Special Pathology and Treatment of Internal Diseases. By Christfried Jakob. Authorized translation from the German. Edited by Augustus A. Eshned, M. D.

182 colored illustrations upon 68 plates, and 64 illustrations in the text. Published by W. B. Saunders, Philadelphia.

This work has had such a deserved popularity in Germany that we do not hesitate to predict a like reception among the English-speaking races. The first part of the book is devoted to full-page colored charts picturing the chemical and microscopical reactions of pathological processes, while the latter half discusses in an outline manner the methods of general and special diagnosis.

The text is concise, but full, clear and to the point. The illustrations are excellent, from an artistic standpoint, and are true and faithful in detail. It is, on the whole, a very fine treatise and needs no better recommendation than to be seen and used.

